# Artificial Generation of High Harmonics via Nonrelativistic Thomson Scattering in Metamaterial

**DOI:** 10.34133/2019/8959285

**Published:** 2019-02-07

**Authors:** Yongzheng Wen, Ji Zhou

**Affiliations:** State Key Laboratory of New Ceramics and Fine Processing, School of Materials Science and Engineering, Tsinghua University, Beijing 100084, China

## Abstract

High harmonic generation allows one to extend the frequency of laser to a much broader regime and to study the electron dynamics of matters. However, severely limited by the vague high-order process in natural material and the unfriendly state of the commonly applied gas and plasma media, the ambitious goal of custom-design high harmonics remains exceptionally challenging. Here, we demonstrate that high harmonics can be artificially designed and tailored based on a metamaterial route. With the localized reconstruction of magnetic field in a metamaterial, the nonlinear Thomson scattering, a ubiquitous electromagnetic process which people used to believe that it only occurs with the relativistic velocity, can be stimulated in a nonrelativistic limit, which drives anharmonic oscillation of free electrons and generates high harmonics. An explicit physical model and the numerical simulations perfectly demonstrate the artificial generation and tailoring of the high harmonics. This novel mechanism is entirely dominated by the artificial structure instead of the natural nonlinear compositions. It not only provides unprecedented design freedom to the high harmonic generation but breaks the rigorous prerequisite of the relativistic velocity of the nonlinear Thomson scattering process, which offers fascinating possibilities to the development of new light source and ultrafast optics, and opens up exciting opportunities for the advanced understanding of electrodynamics in condensed matters.

## 1. Introduction

Thomson scattering, one of the most fundamental mechanisms in electrodynamics, is the elastic photon-electron scattering under moderate intensity electromagnetic (EM) radiation [1], which is a linear process and does not change the frequency of light, while, for nonlinear Thomson scattering with the ultrahigh intensity laser, the free electrons oscillate with the drift velocity approaching the vacuum speed of light, and the contributions of magnetic and electric fields of light to the Lorentz force become comparable, consequently leading to anharmonic motion of the electron and various nonlinear phenomena [2–5]. One of them is the high harmonic generation (HHG), which is overwhelmingly favorable due to its critical potential in extending the frequency range of the table-top laser system, and the ultrafast dynamics [6–9]. However, due to the inevitable requirement of relativistic velocity, almost all the researches of nonlinear Thomson scattering focus on plasma electrons, making the artificial engineering of high-order nonlinearity tremendously difficult. In fact, besides the nonlinear Thomson scattering, most currently available HHG mechanisms are based on gas and plasma [10, 11], and they all suffer from the similar plight. Although some research reveals that several specific solid-state crystals and graphene can generate high harmonics under the extremely strong field illuminations and present primitive controllability [12–17], it is still far away from the ambitious goal of precisely predicting, custom design, and exact tailoring of the high harmonics, especially considering the essential mechanism of the HHG in materials is still vague and the proposed physical models are basically qualitative and semiquantitative [14, 18–21].

Metamaterial may be a promising concept for realizing the artificial HHG, since its unprecedented degree of design freedom has been extensively demonstrated in linear optics with a variety of extraordinary properties [22–25]. Several very recent studies demonstrate that by incorporating with micro/nanostructures, such as metamaterial and plasmonics [16, 26–28], the properties of the HHG media can be manipulated to a certain degree. Nevertheless, a basic fact is that the high-order responses in virtually all the reported structures are inherently from the natural nonlinear materials or nonlinear devices, which cannot fulfill the desire of artificially designing and rationally tailoring the HHG because the artificial structures only play roles of enhancement.

In this work, we propose an entirely artificial mechanism for generation of high harmonics based on a nonrelativistic nonlinear Thomson scattering process in a solid-state metamaterial. By locally redistributing the magnetic field in a metamaterial, the magnetic force becomes comparable to the electric one with a nonrelativistic velocity of free electrons in solids, which generates high harmonics without involving any external nonlinear materials. Numerical simulations perfectly verify the artificial generation of high harmonics, and the geometric impacts on it are studied as well to demonstrate the ultrahigh design freedom.

## 2. Results

### 2.1. Artificial Mechanism

**Figure 1:**
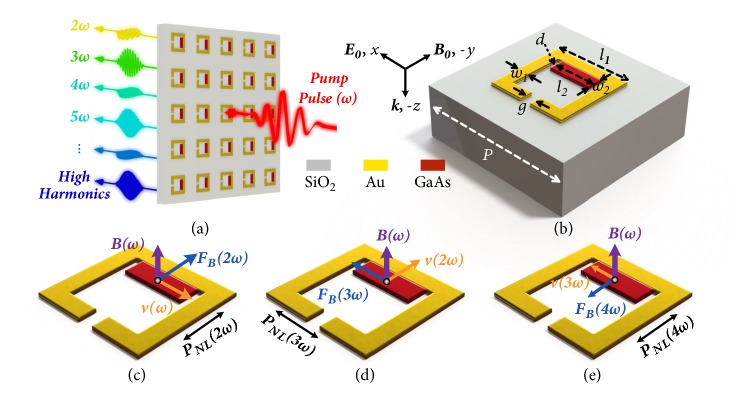
**Metamaterial for artificial high harmonic generation.** (a) Schematic of metamaterial with the polarization state of harmonics indicated. (b) Unit cell of metamaterial. In terahertz regime, the geometric constants are as follows:* P*=30 *μ*m,* l*_*1*_=15.6 *μ*m,* w*_*1*_=2.5 *μ*m,* w*_*2*_=3 *μ*m,* l*_*2*_=8.6 *μ*m,* g*=2 *μ*m, and* d*=1 *μ*m. The relations of the localized magnetic field* B(ω)*, the drift velocity of the free electrons* v(ω)*, and the magnetic force* F*_*B*_*(ω) *for 2^nd^ (c), 3^rd^ (d), and 4^th^ (e) harmonics with the corresponding orientations of the polarizations marked.

Conventionally, the relativistic velocity of the free electron plays a prerequisite role in the nonlinear Thomson scattering, which guarantees the evident magnetic force. In the nonrelativistic regime, however, the essential magnetic force is neglected in a majority of materials because the drift velocity is far slower than the speed of light, and the intrinsic magnetic field from the EM wave is negligibly weak. Meanwhile, the localized magnetic field redistribution of the metamaterial at resonance has primarily been acknowledged [29, 30]. Given these facts, we can then make a rational conception that the exceptionally strong magnetic field, which can be achieved by the local field reconstruction of metamaterial, would realize the nonlinear Thomson scattering process with nonrelativistic motion of free electrons and further artificially generate the high harmonics. Based on the theoretical conception, a metamaterial was designed as depicted in Figures 1(a) and 1(b), and the unit cell consists of a cut-wire resonator nested in a split-ring resonator (SRR).

With an* x*-polarized EM wave normally incident along* z* axis, a magnetic field localized inside the SRR would be stimulated by circulating surface currents at resonance, which is perpendicular to the metamaterial plane and can be potentially enhanced by hundreds of times compared to the magnetic field of the incident wave [29]. Driven by the local electric field, the free electrons in the cut-wire resonator would drift in* x*-direction, and a strong magnetic force would be generated as it locates inside the SRR. Since the local magnetic field does not uniformly distribute inside the SRR, to make the best of the enhancement without breaking the resonance, the cut-wire resonator attaches to the bottom bar of the SRR, where the maximum amplitude is presented. With the drift velocity of free electrons at the fundamental frequency ${\overset{⃑}{v}}_{1}$, the magnetic force could be expressed as

(1) F⃑B2qv⃑1×B⃑=qμ~eωE⃑ωB⃑ωe−i2ωta^y+c.c.,

where ${\tilde{\mu }}_{e}(\omega )$ is the free electrons mobility of the cut wire at angular frequency* ω*, $\overset{⃑}{B}$ is the local magnetic field with $\overset{⃑}{B}(\omega )$ as its vectorial amplitude, $\overset{⃑}{E}(\omega )$ is the vectorial amplitude of the local electric fields,* t* is time,* q* is elementary charge, ${\hat{a}}_{y}$ is the unit vector along the* y* axis, and* c.c.* is complex conjugate. For clarity, the complex conjugate is eliminated in the following calculation. The exhibited second-order term of the magnetic force would drive the free electrons in* y* direction at doubled frequency with the velocity ${\overset{⃑}{v}}_{2}$. Further, the existence of the second-order motion would induce a third-order magnetic force under the localized magnetic field. Different from the second-order term, the third-order magnetic force would polarize along the* x* axis. Similarly, we can in principle continue along these processes, leading to a fourth-order magnetic force along* y* axis, which then leads to a fifth-order one along* x* axis, etc. The physical models of 2^nd^- to 4^th^-order magnetic force are illustrated in Figures 1(c)–1(e), respectively, as examples.

As the magnetic force is dominated by the localized fields, which arise from the resonance between the metamaterial and the incident EM fields, their amplitudes can be described as $\left|\overset{⃑}{B}(\omega )\right|=u(x,y,z)\left|{\overset{⃑}{B}}_{0}(\omega )\right|$ and $\left|\overset{⃑}{E}(\omega )\right|=v(x,y,z)\left|{\overset{⃑}{E}}_{0}(\omega )\right|$, where ${\overset{⃑}{B}}_{0}(\omega )$ and ${\overset{⃑}{E}}_{0}(\omega )$ are the vectorial amplitude of the incident magnetic and electric fields, respectively, and* u(x,y,z)* and* v(x,y,z)* are the enhancement coefficients for the magnetic and electric fields at the coordinate* (x,y,z),* respectively. The N^th^-order magnetic force can thus be derived as

(2) F⃑BNqcN−1∏k=1N−1μ~ekωvx,y,zuN−1x,y,z·E⃑0ωNe−iNωta^y||a^x.

where the relation of $\left|{\overset{⃑}{E}}_{0}(\omega )\right|=\left|{\overset{⃑}{B}}_{0}(\omega )\right|/c$ is considered,* c* is the speed of light in vacuum, and ${\hat{a}}_{x}$ is the unit vector along the* x* axis. For the even order, the magnetic force is along* y* axis, and, for the odd order, it is along* x* axis. The sufficiently enhanced magnetic force would guarantee the occurrence of the nonlinear Thomson scattering process in a nonrelativistic limit and the generation of the high harmonics, which is essentially dominated by the structure of the metamaterial through the enhancement coefficients. As indicated in (2), the metamaterial can provide HHG in both even and odd orders without involving the anisotropic material, and their polarization states are orthogonal. With the induced magnetic field along* z* axis, the magnetic force oscillates the free electrons of the cut-wire resonator in the* xoy* plane, and the HHG waves are expected to radiate along both* +z* and* –z* directions. More importantly, this HHG intrinsically originates from the universally existing magnetic force rather than some particular property of the composites, offering unprecedented freedom of artificial design and manipulation.

To explicitly describe the high-order behavior of the metamaterial, we studied its equivalent susceptibility at different orders. Since the process occurs in a solid structure, the simple motion formula of the conventional Thomson scattering based on the plasma electron is not applicable, and the collisions should be considered. We thus modified the classical Drude model by substituting the magnetic force (see (2)), and the N^th^-order motion of the free electrons can be described as

(3) m∗d2r⃑Ndt2+m∗γdr⃑Ndt=qcN−1·∏k=1N−1μ~ekωvx,y,zuN−1x,y,zE⃑0ωN·e−iNωta^y||a^x,

where *m*^*∗*^ is the effective electron mass, *γ* is the electron collision rate, and ${\overset{⃑}{r}}_{N}$ is the N^th^-order displacement from the equilibrium position. As the polarization of the material is in fact the density of the dipole moments, the general formula for the N^th^-order susceptibility can be deducted as

(4) $$\begin{array}{c} \chi^{\left(N\right)}\left(\;\omega \;\right)=-\frac{\omega_{p}^{2}}{c^{N-1}}\overset{N-1}{\underset{k=1}{\prod }}{\tilde{\mu }}_{e}\left(\;k\omega \;\right)G\left(\;N\omega \;\right)u_{avg}^{N-1}v_{avg}, \end{array}$$

where

(5) $$\begin{array}{c} G\left(\;N\omega \;\right)=\frac{1}{{\left(N\omega \right)}^{2}+iN\omega \gamma }, \end{array}$$

*u*_*avg*_ and* v*_*avg*_ are the average values of the* u(x,y,z) *and* v(x,y,z),* respectively, and* ω*_*p*_ is the plasma frequency of the material forming the cut-wire resonator (mathematical details are described in Supplementary Materials). It can be found that the enhancement coefficients are the key factors determining the high-order nonlinear susceptibility, which are solely defined by the artificial geometry of the metamaterial. Therefore, by elaborately varying the structure, the high-order nonlinearity generated from the metamaterial can be designed and controlled at will.

Several characteristics of the artificial HHG can be predicted from (4). In the first place, the efficiency of the harmonics from the metamaterial would be substantially enhanced, comparing with that from the conventional nonlinear Thomson scattering. It is because the artificial nonlinear process occurs in the solid-state cut-wire resonator instead of the plasma, and the plasma frequency of the solid is orders of magnitude higher than that of the plasma due to the much denser free electrons. In addition, the localized enhancement of the magnetic field apparently improves the HHG. Secondly, the descent of the* G(Nω)* and mobility with the frequency will lead to a monotonic decreasing efficiency of the harmonics with the increasing order. This phenomenon is expected due to the perturbative properties of the magnetic force, which fundamentally distinguishes our theory from the nonperturbative HHG in most natural materials with the plateau region and harmonic cutoff exhibiting in the spectrum.

High conductivity of the SRR would improve the intensity of the HHG, because its characteristic of low loss increases the current density and provides stronger localized magnetic field ([Supplementary-material supplementary-material-1]). Another factor that may influence the HHG is the mobility of the cut-wire resonator. Although the mobility at different frequency varies due to the dispersion, a common term is the dc mobility* μ*_*e0*_. Therefore, by further analyzing (4), the proportional relation between the N^th^-order susceptibility and (*μ*_*e0*_)^*N -1*^ can be obtained. The physical essence is easy to understand that the high mobility leads to fast drift velocity, strong magnetic force, and intense HHG. For the cut-wire resonator, high conductivity usually means high density of free electrons, which would not only induce much more frequent collision and dramatically decrease the mobility but shield the local magnetic and electric field. Both are negative factors and may weaken the HHG.

### 2.2. Illustrative Metamaterial

To verify the artificial mechanism of HHG, we optimized the metamaterial shown in Figure 1 to response in terahertz (THz) regime, and the structure was modeled and simulated by a commercial finite-element package (COMSOL Multiphysics). The substrate was 10 *μ*m thick SiO_2_. Guided by the theory, the SRR is comprised of a 500 nm thick gold, and a 500 nm thick n-doped GaAs film is modeled as the cut-wire resonator due to its high mobility. The details of the materials and simulation settings are described in the Materials and Methods. All compositions are treated as linear materials in the simulation, and they are chosen solely due to their linear properties, such as mobility and conductivity. The metamaterial could be composed of any material that satisfies the fundamental requirements of the basic principle. It should also be noted that, despite the illustration of the mechanism in THz regime, this theory of artificial HHG can be easily extended to other frequencies by simply scaling the geometry of the metamaterial.

**Figure 2:**
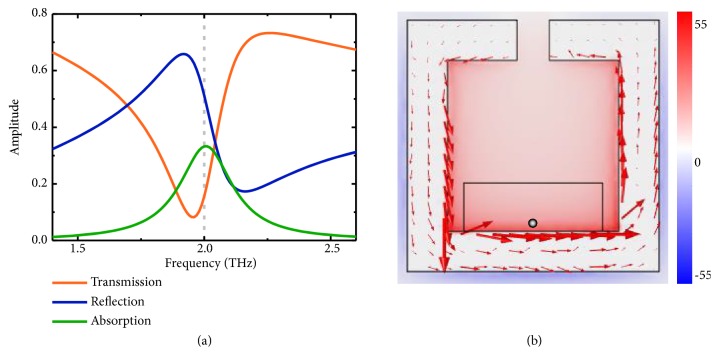
**Linear characteristics of the THz metamaterials.** (a) Transmission, reflection, and absorption spectra of the metamaterial with 2.0 THz marked with grey dashed line; (b) the magnetic field distribution with the scale normalized to the incident magnetic field amplitude and the orientation of the surface currents marked with red arrows.

We first simulated the linear response of the metamaterial at the frequency domain to study its resonant behavior.  Figure 2(a) reveals the transmission, reflection, and absorption spectra of the metamaterial under normal incidence. A resonance peak at 2.0 THz can be observed, where the localized magnetic field reaches the maximum. The surface currents and magnetic distribution at 2.0 THz in Figure 2(b) demonstrate that the circulating currents produce the enhanced magnetic field 62.4 times as intense as the incident one, which are significant enough to generate high harmonics. The existence of the cut-wire resonator only has slight impact on the magnetic field distribution, and the resonant behavior of the SRR remains. The induced magnetic field can penetrate through the cut wire due to its thinness.

**Figure 3:**
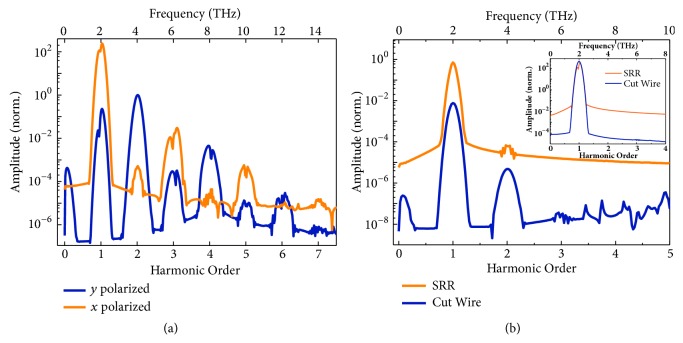
**Transmitted high-harmonic spectra.** (a) Transmitted high-harmonic spectra of the metamaterial in* y* and* x* polarizations; (b) the transmitted high-harmonic spectra of the SRR and cut-wire resonators in* y* polarization; the inset is those in* x* polarization. The amplitudes are normalized to the transmitted second harmonic in* y* polarization generated by the metamaterial.

With a Gaussian pulsed plane wave at 2.0 THz incident from the top, the temporal response of the metamaterial was then simulated. The peak amplitude of the incident electric field was set as 1×10^7^ V/m, corresponding to the power density of 1.3×10^7^ W/cm^2^, which is orders of magnitude lower than the general requirement of the conventional nonlinear Thomson scattering (~10^18^ W/cm^2^) [2, 5] and can be easily achieved with a current-available table-top THz laser [28, 31, 32]. As shown in Figure 3(a), we examined a* y* polarized transmission spectrum in frequency domain, which is transformed from the time response of the metamaterial by Fourier transformation ([Supplementary-material supplementary-material-1]). The spectrum exhibits distinct peaks at both even and odd multiples of the fundamental frequency, corresponding to 0^th^ to 7^th^ harmonics, adequately demonstrating the artificial generation of high harmonics from the metamaterial. Due to the perturbation, the intensity of the harmonic wave decays with the increasing order. The peak at 0^th^ order is the optical rectification signal from the 2^nd^-order nonlinearity. The 2^nd^ harmonic possesses the strongest electric field of 2.6×10^4^ V/m, and the peak value at 6^th^ harmonic is 0.38 V/m. We also investigated the transmission spectrum in* x* polarization, and the harmonic peaks up to 7^th^ order are apparent in Figure 3(a) as well. The amplitude of the 7^th^ harmonic is 0.16 V/m. The amplitudes of each harmonic are listed in [Supplementary-material supplementary-material-1]. Comparing the two spectra, it can be identified that the even order harmonics are generally polarized along* y* direction, while the odd order ones are polarized along* x* direction, which will be further discussed below. All these results are in perfect agreement with the theoretical prediction.

We also simulated the metamaterials containing only SRR or cut-wire resonator for comparison, and the same Gaussian plane wave at 2.0 THz illuminated from the top. Figure 3(b) plots the transmitted spectra of the resonators in* y* and* x* polarization. There are only very weak second harmonic generation observed from the SRR and 2^nd^ and 3^rd^ harmonics from the cut-wire resonator, which are totally negligible compared to those of the metamaterial. No HHG is detected in neither of the resonators, not in* x* or* y* component of the transmitted electric field.

**Figure 4:**
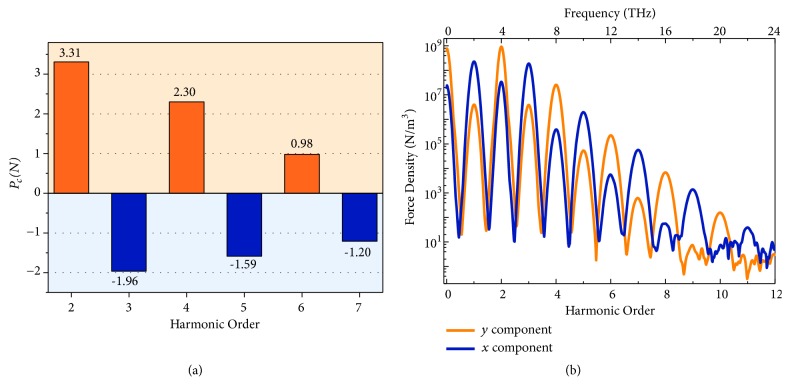
**Polarization of the generated high harmonics.** (a) The* P*_*c*_*(N)* values at each order to indicate the polarization state of harmonics; (b) the harmonic spectra for the* y* and* x* components of the magnetic force at the point marked in Figure 2(b).

To further illustrate the parity of the harmonic order can be distinguished by the polarization state, we defined a parameter* P*_*c*_*(N)* = lg*(E*_*y*_*(N)/E*_*x*_*(N))*, where* E*_*y*_*(N)* and* E*_*x*_*(N)* are the* y* and* x* components of the transmitted peak electric fields at N^th^ order, respectively. As exhibited in Figure 4(a), the even (odd) harmonics present positive (negative) value of* P*_*c*_*(N)*, suggesting that the primary polarizations of the even (odd) harmonics are in* y (x)* direction. Because the magnetic force becomes relatively weaker at high order, the oscillation direction of the free electrons is not rigorously along the* x* or* y* direction, resulting in that the polarization angles of the high orders are not as perfect as those of the low orders. However, it should be noted that the absolute value of* P*_*c*_*(N)* > 1 corresponds to an extinction ratio over 10:1, which is sufficiently high to predominate the polarization state. For example, with respect to the* x* axis,* P*_*c*_*(6*) = 0.98 of the 6^th^ order corresponds to the polarization angle of 84.0°, and* P*_*c*_*(7*) = -1.2 of the 7^th^ order corresponds to that of 3.6°.

We probed the* x* and* y* components of the magnetic force density at the point marked in Figure 2(b) to verify the physical essence of HHG. The magnetic force density in Figure 4(b) presents clear peaks at all the harmonic frequencies in both* x* and* y* components, resembling the conventional nonlinear Thomson scattering. At even orders, the magnetic force in* y* direction dominates the motion of the free electrons, while, at odd orders, that in* x* direction overwhelms. It proves that the generation and parity-dependent polarization of the high harmonics are the macroscopic phenomena of the nonlinear Thomson scattering in a nonrelativistic limit. The maximum order of the magnetic force is 11, higher than that of the HHG, which indicates the metamaterial is potentially capable of generating higher order harmonics by further optimizing the structure or increasing the incident power (see [Supplementary-material supplementary-material-1] and [Supplementary-material supplementary-material-1]).

**Figure 5:**
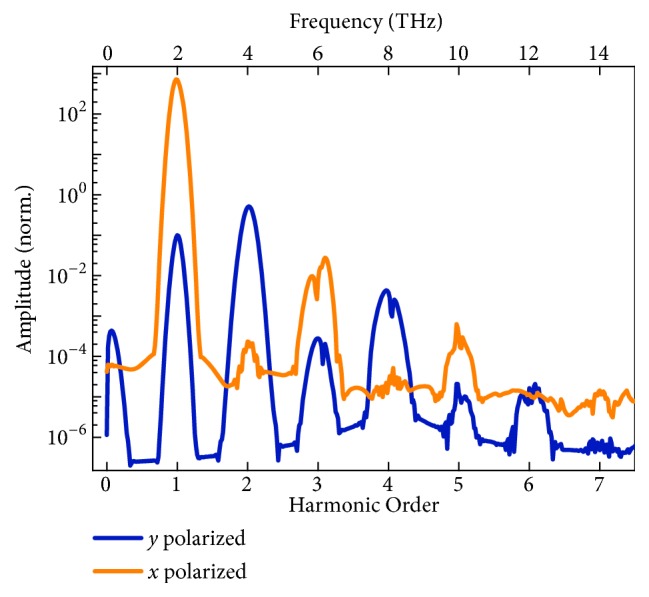
**Reflected high-harmonic spectra of the metamaterial in* y* and* x* polarizations.** The amplitudes are normalized to the transmitted second harmonic in y polarization generated by the metamaterial.

As specified in the theory, the proposed metamaterial radiates longitudinally in both directions, and the reflected spectra are thereby examined as shown in Figure 5 with the evident HHG signals up to 7^th^ order. The peak electric field of 2^nd^ harmonic in* y* polarization is 1.3×10^4^ V/m, and that of the 7^th^ harmonic in* x* polarization is 0.14 V/m. The amplitudes of each harmonic are listed in [Supplementary-material supplementary-material-1]. The same as the emitted HHG in transmission mode, its parity can be separated by the polarization state.

**Figure 6:**
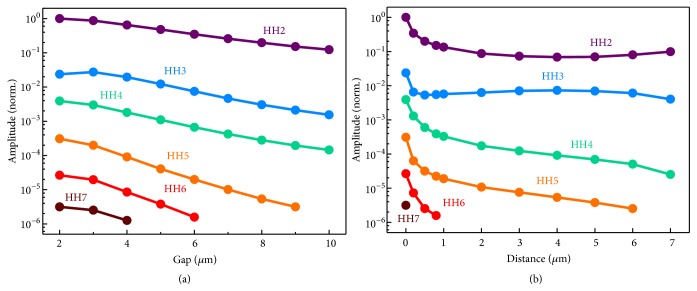
**Geometrical influences on the amplitudes of the transmitted HHG.** (a) The gap of the SRR; (b) the distance between two resonators. The amplitudes are normalized to the transmitted second harmonic in* y* polarization generated by the metamaterial.

To confirm the artificial designability and tailoring of the high harmonics generated from the metamaterial, we manipulated the geometry of the unit cell and studied the influence on the amplitude of the HHG. For clarity and better contrast, only the dominant electric field components of the transmitted harmonics have been studied, namely,* y* component for the even orders and* x* for the odd ones. The gap of the SRR was firstly varied. With the gap widening, the resonant frequency shifts, and the local magnetic field at 2.0 THz weakens rapidly ([Supplementary-material supplementary-material-1]), thus leading to decreasing harmonic amplitude, as shown in Figure 6(a). Some high-order harmonics even vanish due to the too wide gap.

The other geometric characteristic we studied is the location of the cut-wire resonator. By tailoring the distance between cut wire and bottom of the SRR from 0 (attached) to 7 *μ*m, the HHG amplitudes are compared in Figure 6(b). Since the magnetic field distributes nonuniformly inside the SRR, the existence of the distance would significantly weaken the magnetic force, leading to an abrupt drop of the harmonics intensities ([Supplementary-material supplementary-material-1]). For the harmonics over 4^th^ order, the large distance leads to a monotonic decay of the amplitude, and the 7^th^-order signal only exists at 0 distance. For the 2^nd^ harmonic, the amplitude first drops and then rises with increasing distance, while for the 3^rd^ harmonic, except the point at 0, the amplitude first rises and then drops. By interpreting (4), we can find that, at lower order, the role of the magnetic and electric fields in the nonlinear polarization is comparable, while, at high order, the weight of the magnetic field is absolutely superior to that of the electric field. Therefore, although the large distance results in weak magnetic field, it locates the cut wire close to the gap of the SRR, where the electric field is considerably enhanced, making the variation trend of the 2^nd^ and 3^rd^ harmonics nonmonotonic, while, at high harmonics, the weak magnetic field due to the large distance cannot be compensated by the enhancement of the electric field, leading to the monotonic decreasing behavior.

Inspired by these two examples, it can be conveniently predicted that other geometric constants would also have an effective impact on the high-order nonlinear response, including the width (*w*_*1*_) and length (*l*_*1*_) of the SRR, and the lattice constant of the metamaterial (*P*). The artificial tailoring offers the HHG not only the high design freedom, but also the potential of the real-time manipulation by combining with the active modulation techniques, such as microelectromechanical system (MEMS) and photostrictive materials [33, 34].

All these simulated results profoundly demonstrate the proposed artificial mechanism of HHG without involving any sophisticated mesoscopic and quantum mechanisms in materials or external nonlinear insertions. The HHG essentially arises from the fundamental nonlinear Thomson scattering in a nonrelativistic limit, which is dominated by the artificial metamaterial structure instead of the natural compositions, making the method available to almost all the conducting materials, such as doped silicon and graphene, not limited to the GaAs (details are described in [Supplementary-material supplementary-material-1] and Supplementary Materials).

The HHG from the metamaterial contains both even and odd orders, and the parity can be distinguished by the polarization state, which is in sharp contrast to the majority of natural isotropic media, including the commonly illustrated semiconductor crystal and rare gas, which can only generate odd order harmonics because of the symmetry inversion [27, 35]. In the meantime, the “phase matching” condition for the artificial HHG is different from the conventional nonlinear optics, as it is dominated by the artificial structure rather than any special nonlinear property of the natural materials. The commonly used phase-matching methods, such as oblique incidence and configuring the polarization of the incident wave, may have negative impacts on the resonance and magnetoelectric coupling behaviors of the metamaterial and erodes the efficiency. Considering the fact that the HHG efficiency of the metamaterial reaches maximum with the conditions of the normal illumination and linear polarization, it is appropriate to treat them as equivalent phase matching conditions for the artificial harmonics generation. It should also be noted that although only harmonics generation is demonstrated, the wave mixing effects can occur in the proposed metamaterial as well. As presented in Figure 2(a), the resonance bandwidth of the metamaterial is relatively broad. With the incident EM wave containing multiple frequencies within the resonance bandwidth, the metamaterial can simultaneously respond to all the fundamental frequencies, and the coupling between each frequency will lead to the generation of sum and difference frequency.

Since the proposed artificial mechanism provides an approach of achieving the nonlinear Thomson scattering in solid-state materials with no request of relativistic velocity, the demand of the intense illumination of the light would be substantially relaxed. To quantitatively verify it, we compare the amplitude of the normalized vector potential in the definition form of $\alpha_{0}=\left|{\overset{⃑}{F}}_{B}\right|/\left|{\overset{⃑}{F}}_{E}\right|$, and it is equivalent to the classical form $\alpha_{0}=q\left|{\overset{⃑}{E}}_{0}(\omega )\right|/m_{0}\omega c$ for conventional nonlinear Thomson scattering [3, 6], where* m*_*0*_ is the electron mass. In the metamaterial, the *α*_*0*_ of the free electrons in the cut-wire resonator can be theoretically calculated as 0.75, which is 1.6×10^3^ times stronger than that of the plasma electrons, corresponding to 2.6×10^6^ times higher power density of the incident laser (calculation details are described in Supplementary Materials). This significant enhancement overcomes the longstanding obstacle of the near-light speed in the study of the nonlinear Thomson scattering and makes its occurrence in low velocity and in condensed phase material possible. This novel mechanism may become a generic tool to advance the understanding of the fundamental electrodynamics of the condensed matters. Given the fact that the proposed design is only a proof-of-concept, we believe, by optimizing the structure of the metamaterial, the nonlinear scattering would be further improved.

More importantly, by simply engineering the geometry of the metamaterials, the artificially generated high harmonics can be precisely designed and exactly tailored with ultrahigh degree of freedom. Although the metamaterial is numerically demonstrated in THz regime, due to the universal existence of the Lorentz force, this theory of artificial high harmonics can be easily extended to wide wavelength ranging from microwave to infrared and visible light by properly scaling the metamaterial (details are described in Figures [Supplementary-material supplementary-material-1] and [Supplementary-material supplementary-material-1], [Supplementary-material supplementary-material-1], and Supplementary Materials). In the meantime, the metamaterial based on the nonrelativistic nonlinear Thomson scattering is highly achievable in practice, like most reported metamaterial for other applications [24, 25, 36, 37]. The metamaterials designed for the long wavelength regime, such as microwave and THz, could be conveniently manufactured by microfabrication techniques, including the standard ultraviolet lithography, magnetron sputtering, chemical vapor deposition (CVD), and molecular beam epitaxy (MBE). Those for the short wavelength regime, such as infrared and visible light, could be processed by nanoengineering methodology based on the electron-beam lithography and focused ion beam (FIB) technology ([Supplementary-material supplementary-material-1]). The independence of this artificial mechanism on the compositions allows choosing the fabrication-friendly materials to form the metamaterial, further guaranteeing its practical realization.

These fascinating features of the proposed mechanism would open groundbreaking possibilities and tons of novel phenomena to the high-order optical nonlinearity, metamaterial, ultrafast physics, and electrodynamics of matters. For instance, the active modulation and feedback tuning of the high harmonics, and the flat self-focusing lens and holography for high harmonics, and compact attosecond laser might all become achievable.

## 3. Discussion

We theoretically demonstrated an artificial mechanism for high harmonic generation based on a nonrelativistic Thomson scattering in a metamaterial. As adequately described by an explicit physical model, the locally reconstructed magnetic field in the metamaterial stimulates the nonlinear Thomson scattering in solid with a nonrelativistic velocity, which drives the free electrons to oscillate in an anharmonic way and further generates the high harmonics. A series of numerical simulations perfectly support the proposed theory with the artificial generation of high harmonics, and the geometric variation of the metamaterial leads to an apparent control and tailoring. This purely artificial mechanism provides extraordinary degree of design freedom to high harmonic generation with a metamaterial-based approach and allows the occurrence of nonlinear Thomson scattering in a nonrelativistic limit, which would open myriad application possibilities and novel potentials to high-order nonlinearity and advanced understanding of the electron dynamics in condensed matters.

## 4. Materials and Methods

### 4.1. Materials Modelling

In THz regime, the substrate was SiO_2_ with the permittivity of 4.2+0.026*i*. The gold layer of the SRR was modeled with the conductivity of 4.1×10^7^ S/m and mobility of 29.5 cm^2^/V•s [38, 39]. A n-doped GaAs film is modeled as the cut-wire resonator with free electron density of 5×10^17^ cm^−3^, corresponding to the dc conductivity of 3.1×10^4^ S/m and the dc mobility of 3800 cm^2^/Vs with the damping frequency of 2*π*×6.4 THz, and the permittivity at high frequency is 12.9 [40]. With the existence of the locally enhanced magnetic field, the GaAs film was modeled with an anisotropic Drude conductivity tensor, as follows [41, 42]:

(6) $$\begin{array}{c} \tilde{\sigma }\left(\;\omega \;\right)=\tilde{\sigma }\left[\begin{array}{ccc} \frac{1}{1+\beta^{2}} & -\frac{\beta }{1+\beta^{2}} & 0\\ \frac{\beta }{1+\beta^{2}} & \frac{1}{1+\beta^{2}} & 0\\ 0 & 0 & 1 \end{array}\right], \end{array}$$

where $\beta ={\tilde{\mu }}_{e}(\omega )\left|\overset{⃑}{B}(\omega )\right|$, $\tilde{\sigma }=\sigma_{0}/\left(1-i\omega /\gamma \right)$, and ${\tilde{\mu }}_{e}(\omega )=\mu_{e0}/\left(1-i\omega /\gamma \right)$,* σ*_*0*_ and* μ*_*e0*_ are the dc conductivity and mobility, respectively, and *γ* is damping frequency. In the control simulation, the gold of the SRR was modeled with the same anisotropic conductivity tensor.

### 4.2. Simulation Settings

The metamaterial was simulated in both frequency and time domains. In both domains, single unit cell was simulated with the periodic boundary condition in* x* and* y* directions, and the ports for transmitting and receiving wave were set on the top and bottom boundaries, respectively. The localized magnetic field was incorporated by simply inputting the variable representing the local magnetic field when defining the anisotropic materials.

In frequency domain, the S-parameters of the metamaterial were simulated ranging from 1.4 THz to 2.6 THz with the step of 0.01 THz. Therefore, the reflection (*R*), transmission (*T*), and absorption (*A*) spectra can be obtained with the relations *R* = |*S*_11_|^2^, *T* = |*S*_21_|^2^, and* A=1-T-R*.

In time domain, the* x*-polarized incident wave was defined by the electric filed ${\overset{⃑}{E}}_{0}=\left({\overset{⃑}{E}}_{x},0,0\right)$, and ${\overset{⃑}{E}}_{x}$ is

(7) $$\begin{array}{c} {\overset{⃑}{E}}_{x}={\overset{⃑}{E}}_{0}\left(\;\omega \;\right)\cos\left(\;\omega t+k_{0}z\;\right)e^{-{\left(\left(t-t_{0}\right)/\Delta t\right)}^{2}}, \end{array}$$

where ${\overset{⃑}{E}}_{0}\left(\omega \right)$ is the peak amplitude of the electric field,* ω *is the angular frequency,* t* is time,* k*_*0*_ is the wavenumber at the frequency* ω*,* z* is the coordinate in* z* axis, and* t*_*0*_ and* Δt* are the parameters describing the Gaussian pulse. The values used in the time-domain simulations are $\left|{\overset{⃑}{E}}_{0}\left(\omega \right)\right|$=10^7^ V/m,* ω*=2*π*×2×10^12^ rad/s,* t*_*0*_=7 ps, and* Δt* =2 ps. The total time of 30 ps was simulated with the step of 2 fs.

## Supplementary Materials

Supplementary Materials Figure S1: the influence of conductivity of SRR on the metamaterial. Figure S2: transmission spectra in the time domain of the metamaterial. Figure S3: transmitted high-harmonic spectra of the metamaterial in* y* and* x* polarizations. Figure S4: characteristics of metamaterial with different gap. Figure S5: spectra of the total magnetic force on the cut-wire resonator with different distance between two resonators. Figure S6: transmitted high-harmonic spectra of the metamaterial with doped Si as the cut-wire resonator in y and x polarizations. Figure S7: transmission, reflection, and absorption spectra of the infrared metamaterial with 30 THz marked with grey dashed line. Figure S8: transmitted high-harmonic spectra of the infrared metamaterial in* y* and* x* polarizations. Figure S9: an exemplary fabrication process of the metamaterial. Table S1: electric fields of all harmonics in transmission. Table S2: electric fields of all harmonics in reflection. Table S3: electric fields of the transmitted harmonics under more intense incidence. Table S4: electric fields of the transmitted harmonics in infrared regime.
