# Supplementary material for: Artificial Generation of High Harmonics via Nonrelativistic Thomson Scattering in Metamaterial
Source: Research (Wash D C). 2019 Feb 7;2019:8959285. doi: 10.34133/2019/8959285 (PMC6750065; doi:10.34133/2019/8959285)
Supplement: Supplementary Materials — Figure S1: the influence of conductivity of SRR on the metamaterial. Figure S2: transmission spectra in the time domain of the metamaterial. Figure S3: transmitted high-harmonic spectra of the metamaterial in y and x polarizations. Figure S4: characteristics of metamaterial with different gap. Figure S5: spectra of the total magnetic force on the cut-wire resonator with different distance between two resonators. Figure S6: transmitted high-harmonic spectra of the metamaterial with doped Si as the cut-wire resonator in y and x polarizations. Figure S7: transmission, reflection, and absorption spectra of the infrared metamaterial with 30 THz marked with grey dashed line. Figure S8: transmitted high-harmonic spectra of the infrared metamaterial in y and x polarizations. Figure S9: an exemplary fabrication process of the metamaterial. Table S1: electric fields of all harmonics in transmission. Table S2: electric fields of all harmonics in reflection. Table S3: electric fields of the transmitted harmonics under more intense incidence. Table S4: electric fields of the transmitted harmonics in infrared regime. [file 8959285.f1.docx]

*Supplementary Materials*

**Artificial generation of high harmonics via nonrelativistic Thomson scattering in metamaterial**

Yongzheng Wen and Ji Zhou^*^

State Key Laboratory of New Ceramics and Fine Processing, School of Materials Science and Engineering, Tsinghua University, Beijing 100084, People's Republic of China.

^*^Corresponding author: zhouji@tsinghua.edu.cn

1. MATHEMATICAL DETAILS

We first modified the classical Drude model by adding the second-order magnetic force to describe the second-order motion of the free electrons as

. (S1)

Each symbol in the equation has been defined in the main text. By solving the Eq. (S1), the drift velocity $\vec{v}_{2}$ can be obtained as

. (S2)

By substituting the relation $\tilde{\mu}_{e}(2\omega)\text{=}\frac{q}{m^{*}\gamma}\frac{1}{1-i(2\omega)/\gamma}$, the drift velocity becomes

. (S3)

Further, the existence of $\vec{v}_{2}$ would induce a third-order magnetic force under the localized magnetic field, which can be expressed as

. (S4)

By substituting the Eq. (S4) to the Drude model, the third-order motion of the free electrons can be described as

. (S5)

The third-order velocity of the drift motion $\vec{v}_{3}$ can be obtained as

. (S6)

The third-order velocity would induce a fourth-order magnetic force along *y* axis. As mentioned in the main text, we can in principle continue along these processes and lead to a N^th^-order magnetic force, which can thus be derived as

. (S7)

By substituting the relation between the localized fields and the incident fields, including $\left|\vec{B}(\omega)\right|\text{=}u(x,y,z)\left|\vec{B}_{0}(\omega)\right|$, $\left|\vec{E}(\omega)\right|=v(x,y,z)\left|\vec{E}_{0}(\omega)\right|$, and $\left|\vec{E}_{0}(\omega)\right|\text{=}\left|\vec{B}_{0}(\omega)\right|/c$, the equation evolves into

, (S8)

which is the Eq. (2) in the main text.

With the magnetic force equation, the N^th^ order motion of the free electrons can be described as

, (S9)

where is the N^th^-order displacement from the equilibrium position. By solving the Eq. (6) with the perturbation method, we can obtain

$\vec{r}_{N}(N\omega)=-\frac{q}{c^{N-1}m^{*}}\prod_{k=1}^{N-1} \tilde{\mu}_{e}(k\omega)v(x,y,z)u^{N-1}(x,y,z)\left|\vec{E}_{0}(\omega)\right|^{N}G(N\omega)\hat{a}_{y}\text{‖}\hat{a}_{x}$, (S10)

where

$G(N\omega)=\frac{1}{(N\omega)^{2}+iN\omega\gamma}$. (S11)

As the localized fields is not uniformly distributed inside the metamaterial, we established the following equation to obtain the N^th^-order polarization vector $\vec{P}_{NL}(N\omega)$

$\vec{P}_{NL}(N\omega)=\frac{qM}{V_{cut}}\int_{V_{cut}} \vec{r}_{N}(N\omega)dV$, (S12)

where *M* is the free electron density, *V_cut_* is the volume of the cut-wire resonator. Thus, $\vec{P}_{NL}(N\omega)$ can be obtained as

$\vec{P}_{NL}(N\omega)=-\frac{\varepsilon_{0}\omega_{p}^{2}}{c^{N-1}}\prod_{k=1}^{N-1} \tilde{\mu}_{e}(k\omega)G(N\omega)u_{_{avg}}^{N-1}v_{_{avg}}\left|\vec{E}_{0}(\omega)\right|^{N}\hat{a}_{y}\text{‖}\hat{a}_{x}$, (S13)

Where *ε_0_* is the vacuum permittivity, and the plasma frequency is

$\omega_{p}=\sqrt{\frac{Mq^{2}}{m^{*}\varepsilon_{0}}}$. (S14)

The *u_avg_* and *v_avg_* are the average values of the *u(x,y,z)* and *v(x,y,z)* respectively. By definition, the N^th^-order polarization can also be expressed as

$\left|\vec{P}_{NL}(N\omega)\right|=\varepsilon_{0}\chi^{(N)}(\omega)\left|\vec{E}_{0}(\omega)\right|^{N}$, (S15)

where $\chi^{(N)}(\omega)$ is the N^th^-order susceptibility. By comparing Eq. (S13) and (S15), we can conveniently acquire the expression of $\chi^{(N)}(\omega)$ as

$\chi^{(N)}(\omega)=-\frac{\omega_{p}^{2}}{c^{N-1}}\prod_{k=1}^{N-1} \tilde{\mu}_{e}(k\omega)G(N\omega)u_{_{avg}}^{N-1}v_{_{avg}}$. (S16)

The Eq. (S11) and (S16) are the Eq. (5) and (4) in the main text, respectively.

1. INFLUENCE OF MATERIAL LOSS ON THE METAMATERIAL

High mobility and low loss materials are preferred in composing the split-ring resonator (SRR) because it would provide more evident resonance and stronger local magnetic field. We thus varied the conductivity of the SRR from 4.1×10^6^ S/m to 2×10^8^ S/m, and the spectra of the maximum local magnetic field in the metamaterial are plotted in Figure S1 (a). The unit cell structure and the methods of simulation are kept the same as described in the main text, and the amplitudes of magnetic field are normalized to that of the 4.1×10^7^ S/m sample, which is the value of Au used in the main text. It can be found that with the decrease of the conductivity, the local magnetic field decreases. It is because the low conductivity results in more frequent collisions between the free electrons and large resistance, which produces more heat and higher ohmic loss.

FIGURE S1: **The influence of conductivity of SRR on the metamaterial. (a)** The spectra of the maximum local magnetic field in the metamaterial. The amplitudes of magnetic field are normalized to that of the 4.1×10^7^ S/m sample. **(b)** The amplitudes of the transmitted HHG, which are normalized to the transmitted second harmonic in *y* polarization generated by the 4.1×10^7^ S/m sample.

Furthermore, we studied the relation of the amplitudes of the transmitted HHG to the conductivity of the SRR as presented in Figure S1 (b). The same plane wave at 2.0 THz with the incident electric field of 10^7^ V/m was used. For clarity, only the dominant electric field components of the transmitted harmonics have been studied, namely *y* component for the even orders and *x* for the odd ones. With the conductivity increasing from 4.1×10^6^ S/m to 2×10^8^ S/m, the amplitudes of HHG increase, and the 7^th^ harmonic only exists in the samples with the conductivity higher than 2×10^7^ S/m. It meets the theoretical expectation that the low loss of the material strengthens the resonance and the localized magnetic field of the metamaterial, which eventually improves the generation of harmonics as indicated in the Eq. (4) in the main text.

It should be noted that despite of the variation in amplitudes, high harmonics can be generated from the metamaterial even with the conductivity of the SRR decreasing by one order of magnitude. Considering the fact that most commonly-available metals, such as Al, Ag and Cu, are in the similar conductivity as Au, we believe the artificial HHG is not limited to some specific high-quality materials.

1. AMPLITUDES OF HIGH HARMONICS

The frequency-domain spectra of the high harmonics shown in the main text are transformed from the time-domain spectra. The overall *y* and *x* components of the transmission spectra in the time domain are plotted in the Figure S2. Further, we can extract the time-domain signals of each harmonic with the band-pass filter, and those of the 2^nd^ and 4^th^ harmonics in y component and the 3^rd^ harmonic in *x* component are revealed examples. Therefore, the peak electric fields of all harmonics in transmission can be obtained, which are listed in Table S1. Similarly, those in reflection can be extracted as well, as listed in Table S2. For clarity, only the dominant values of each harmonic are listed, namely *y* component for the even orders and *x* for the odd ones.

FIGURE S2: **Transmission spectra in the time domain of the metamaterial. (a)** The overall *y* component of the transmission spectra in the time domain, and the band-pass filtered signals of the 2^nd^ and 4^th^ harmonics; **(b)** the overall *x* component of the transmission spectra in the time domain, and the band-pass filtered signals of the 3^rd^ harmonics.

Table S1. Electric fields of all harmonics in transmission

| **Polarization** | **Order** | **Electric Field**  **(V/m)** | **Polarization** | **Order** | **Electric Field**  **(V/m)** |
| --- | --- | --- | --- | --- | --- |
| ***y*** | 2 | 2.6×10^4^ | ***x*** | 3 | 598 |
|  | 4 | 120 |  | 5 | 8.9 |
|  | 6 | 0.38 |  | 7 | 0.16 |

Table S2. Electric fields of all harmonics in reflection

| **Polarization** | **Order** | **Electric Field**  **(V/m)** | **Polarization** | **Order** | **Electric Field**  **(V/m)** |
| --- | --- | --- | --- | --- | --- |
| ***y*** | 2 | 1.3×10^4^ | ***x*** | 3 | 538 |
|  | 4 | 113 |  | 5 | 4.3 |
|  | 6 | 0.28 |  | 7 | 0.14 |

1. POTENTIAL OF GENERATING HIGHER ORDER HARMONICS

To verify that the artificial mechanism can potentially generate even higher order of harmonics instead of the 7^th^ order shown in the main text, we simulated the same metamaterial, but the peak electric field of the incident terahertz (THz) wave was set as 1×10^8^ V/m with the other parameters keeping the same. The total time of 30 ps was simulated with the step of 1 fs. It should be noted that although this electric field is 10 times stronger than that used in the main text, it is still highly practical and achievable because it is more than one order of magnitude weaker than the maximum electric field of the current-available table-top THz laser [1, 2].

We investigated the *y* and *x* polarized transmission spectra in frequency domain of the metamaterial as shown in Figure S3, which are transformed from the time response by Fourier transformation. There are high harmonic signals up to 48 orders observed from both spectra. The highest frequency generated from the metamaterial is 96 THz, corresponding to the wavelength of 3.125 μm, which belongs to the mid-wavelength infrared regime. For the y polarized transmission wave, the 2^nd^ harmonic possesses the highest amplitude of 1.6×10^6^ V/m, and the amplitude of the 48^th^ harmonic is 1.1 V/m. For the x polarized transmission wave, the 3^rd^ harmonic possesses the highest amplitude of 1.5×10^5^ V/m, and the amplitude of the 47^th^ harmonic is 1.1 V/m. With the band-pass filter, the amplitudes of some representative harmonics can be extracted as listed in Table S3. Same as the Sec. III, only the dominant components of the harmonics are listed.

FIGURE S3: **Transmitted high-harmonic spectra of the metamaterial in *y* (a) and *x* (b) polarizations.** The amplitudes are normalized to the transmitted second harmonic in *y* polarization generated by the metamaterial under the incident peak electric field of 10^8^ V/m

Table S3. Electric fields of the transmitted harmonics under more intense incidence

| **Polarization** | **Order** | **Electric Field**  **(V/m)** | **Polarization** | **Order** | **Electric Field**  **(V/m)** |
| --- | --- | --- | --- | --- | --- |
| ***y*** | 2 | 1.6×10^6^ | ***x*** | 3 | 1.5×10^5^ |
|  | 10 | 1.0×10^4^ |  | 9 | 2.3×10^4^ |
|  | 20 | 584 |  | 19 | 586 |
|  | 30 | 91 |  | 29 | 120 |
|  | 40 | 10.2 |  | 39 | 12.5 |
|  | 48 | 1.1 |  | 47 | 1.1 |

1. INFLUENC OF GAP ON THE LOCAL MAGNETIC FIELD

We first studied the resonant behavior of the metamaterial with different gap, and the absorption spectra are plotted in Figure S4 (a). It can be observed that with the increasing gap, the resonant frequency shifts to higher frequency, leading to a weaker resonant strength at 2.0 THz. We further examined the magnetic field distributions of all samples at 2.0 THz, as shown in Figures S4 (b)-(f). The declining strength of the resonance results in the rapid decrease of the localized magnetic field and the magnetic force. Therefore, the intensities of all the harmonics monotonically decrease with the increase of the gap, and the too weak magnetic field causes the disappearance of some high order signals.

FIGURE S4: **Characteristics of metamaterial with different gap.** **(a)** The absorption spectra of the metamaterial with the gap of 2 μm, 4 μm, 6 μm, 8 μm and 10 μm, and 2.0 THz is marked with grey dashed line. The magnetic field distributions at 2.0 THz for the metamaterial with the gap of 2 μm **(b)**, 4 μm **(c)**, 6 μm **(d)**, 8 μm **(e)** and 10 μm **(f)**. The scales are normalized to the incident magnetic field amplitude, and the orientation of the surface currents in the SRR is marked with red arrows.

1. INFLUENC OF ATTACHMENT BETWEEN TWO RESONATORS

We studied the resonant behaviors of the metamaterials with 0 μm, 1 μm and 3 μm distances between two resonators, and the spectra of the total magnetic force on the cut-wire resonator were shown in Figure S5. It can be found that the resonant frequency keeps almost the same with the different distance, but magnetic force for the 0 μm (attached) sample is significantly stronger than the other two. It demonstrates that the tailoring of HHG by changing the position of the cut-wire resonator mainly originates from the nonuniform distribution of the local magnetic field, and the attachment between two resonators only slightly shifts the resonance of the metamaterial, which can be neglected. The attachment can make the best of the induced local magnetic field and provide the strong magnetic force, which eventually generates the HHG.

FIGURE S5: **Spectra of the total magnetic force on the cut-wire resonator with different distance between two resonators.**

1. SIMULATION WITH SILICON AS THE CUT-WIRE RESONATOR

To clarify the artificial generation of high harmonics based on the metamaterial could be applied to other materials not merely the n-doped GaAs, the same structure was simulated in terahertz regime by replacing the material for the cut-wire resonator with n-doped silicon. The silicon was characterized with the doping concentration of 10^17^ cm^-3^, corresponding to the dc conductivity of 10^3^ S/m and dc mobility of 721 cm^2^/Vs, and the damping frequency is 2π×1.4 THz, and the permittivity at high frequency is 11.9 [3]. The anisotropic tensor of the conductivity is also utilized to model the silicon. All the other settings, including the geometry and boundary conditions are kept the same as described in the main text.

Under normal incidence of the 2.0 THz wave, we examined the *x* and *y* polarized transmission spectra in frequency domain as revealed in Figure S6, which is transformed from the time response of the metamaterial by Fourier transformation. Harmonics up to 4^th^ order could be obtained, and the parity of the harmonic order can be distinguished by the polarization state as well. For the *y* polarized transmission wave, the 2^nd^ harmonic possesses the highest amplitude of 76 V/m, and that of the 4^th^ harmonic is 0.04 V/m. The 3^rd^ harmonic in *x* polarization possesses the amplitude of 0.83 V/m. The lower order and weaker amplitude of harmonics than those of the GaAs sample mainly results from the lower mobility of the doped silicon, which has been predicted by the physical model. The simulated results clarify that the proposed theory of the artificial HHG is applicable to a wide variety of conducting material.

FIGURE S6: **Transmitted high-harmonic spectra of the metamaterial with doped Si as the cut-wire resonator in *y* and *x* polarizations.** The amplitudes are normalized to the transmitted second harmonic in *y* polarization generated by the metamaterial.

1. NORMALIZED VECTOR POTENTIAL

As mentioned in the main text, the amplitude of the normalized vector potential is given by $\alpha_{0}=\left|\vec{F}_{B}\right|/\left|\vec{F}_{E}\right|$. Hence, for the nonrelativistic nonlinear Thomson scattering in metamaterial, we could obtain that

. (S17)

As described in the main text, the local magnetic field $\vec{B}(\omega)$ is enhanced by 62.4 times compared to the incident one, and the mobility at angular frequency *ω* is $\tilde{\mu}_{e}(\omega)=\frac{\mu_{e0}}{1-i\omega/\gamma}$. By substituting the values from the main text into the Eq. (S17), including *μ_e0_*=0.38 m^2^/V•s, *E_0_(ω)*=10^7^ V/m, *c*=3×10^8^ m/s, *γ=*2π×6.4 THz, we can calculate the *α_0meta_* value of the metamaterial as 0.75.

For the conventional nonlinear Thomson scattering, the free electron is usually from the plasma, and the formula can then be evolved as

$\alpha_{0pla}=\frac{q\left|\overrightarrow{v}_{0}\right|\left|\vec{B}_{0}(\omega)\right|}{q\left|\vec{E}_{0}(\omega)\right|}\text{=}\frac{\left|\overrightarrow{v}_{0}\right|}{c}$, (S18)

where $\overrightarrow{v}_{0}$ is the drift velocity of free plasma electrons. With the relation $\left|\overrightarrow{v}_{0}\right|\text{=}q\left|\vec{E}_{0}(\omega)\right|/m_{0}\omega$, the Eq. (S18) turns into the classical form $\alpha_{0pla}=q\left|\vec{E}_{0}(\omega)\right|/m_{0}\omega c$, where *m_0_*=9.1×10^-31^ kg is the electron mass. With the above parameters, the *α_0pla_* can be calculated as 4.7×10^-4^. We can then acquire *α_0meta_/α_0pla_ =* 1.6×10^3^. The ratio indicates that, for the plasma electrons, to achieve the same value of 0.75 as the metamaterial, it would require a 1.6×10^3^ times stronger electric field, corresponding to the 2.6×10^6^ times higher power density of the incident laser.

1. SIMULATION IN INFRARED REGIME

To verify the feasibility of the proposed theory of artificial HHG in high frequency regime, we simulated a metamaterial with the same structure as shown in Figure 1 in the main text but working in the infrared regime. The geometric constants are scaled down to *l_1_*= 950 nm, *w_1_*= 180 nm, *g*=120 nm, *l_2_*=510 nm, *w_2_*= 510 nm, *d*=40 nm, and *P*=1500 nm. The substrate was 500 nm thick silicon dioxide with the permittivity of 4.82+0.026*i* in the infrared regime. The SRR is made of 100 nm thick silver, which presents better optical properties than gold, and the cut-wire resonator is also made of 100 nm thick n-doped GaAs. In the infrared regime, the silver film has characterized by Drude model, and the plasma frequency is 2π×2180 THz with the collision frequency of 2π×4.35 THz [4]. The complex anisotropic Drude conductivity tensor is also used to describe the n-doped GaAs.

We firstly simulated the S-parameters of the infrared metamaterial ranging from 25 THz to 35 THz with the step of 0.1 THz. Under the normal illumination of an *x*-polarized plane wave from the top, the reflection, transmission and absorption spectra of the infrared metamaterial were simulated and plotted in Figure S7. The localized magnetic field reaches its maximum at the resonant frequency of 30 THz. The wavelength corresponding to 30 THz is 10 μm, belonging to the infrared regime, where the enhanced magnetic field is as maximum 27.9 times as the incident one.

FIGURE S7: **Transmission, reflection and absorption spectra of the infrared metamaterial with 30 THz marked with grey dashed line.**

The time-domain response of the infrared metamaterial was then simulated with a Gaussian pulsed plane wave at 30 THz casted from the top as well. The parameters for the Gaussian wave are $\left|\vec{E}\left(\omega\right)\right|$=10^8^ V/m, *ω*=2π×30×10^12^ rad/s, *t_0_*=2 ps, *Δt* =600 fs, and the total time of 4 ps was simulated with the step of 1 fs. The peak amplitude corresponds to the power density of 1.3 GW/cm^2^, which can be easily realized by a commercial pulsed infrared laser in practice [5]. We examined both *y* and *x* polarized transmission spectra in frequency domain as plotted in Figure S8, which are transformed from the time response of the infrared metamaterial by Fourier transformation. The spectra exhibit distinct peaks at both even and odd multiples of the fundamental frequency, corresponding to 0^th^ to 10^th^ harmonic, demonstrating the artificial generation of high harmonics in the infrared regime. Due to the more evident dissipation and dispersion in the infrared regime, the highest order of the harmonics generated by the infrared metamaterial is lower than the THz one, but the maximum frequency of the generated 10^th^-order harmonic still reaches 300 THz, corresponding to the wavelength of 1 μm and belonging to the near infrared regime. As predicted by the theory described in the main text, the even order harmonics are polarized along *y* direction, while the odd order ones are polarized along *x* direction. The peak electric fields of all harmonics in transmission are extracted by the band-pass filter as listed in Table S4. For clarity, only the dominant values of each harmonic are listed, namely *y* component for the even orders and *x* for the odd ones.

FIGURE S8: **Transmitted high-harmonic spectra of the infrared metamaterial in *y* and *x* polarizations.** The amplitudes are normalized to the transmitted second harmonic in *y* polarization generated by the infrared metamaterial.

Table S4. Electric fields of the transmitted harmonics in infrared regime

| **Polarization** | **Order** | **Electric Field**  **(V/m)** | **Polarization** | **Order** | **Electric Field**  **(V/m)** |
| --- | --- | --- | --- | --- | --- |
| ***y*** | 2 | 1.6×10^5^ | ***x*** | 3 | 3.3×10^4^ |
|  | 4 | 3.0×10^3^ |  | 5 | 672 |
|  | 6 | 140 |  | 7 | 11.9 |
|  | 8 | 11.7 |  | 9 | 0.98 |
|  | 10 | 0.29 |  | - | - |

1. FEASIBLE FABRICATION PROCESS FOR METAMATERIAL

The metamaterial based on the nonrelativistic nonlinear Thomson scattering is highly achievable in practice. With the currently-available techniques, there would be various methods of manufacturing the metamaterial, and an exemplary fabrication process flow is proposed as schemed in Figure S9. The substrate could choose the material with the high transmission at the target wavelength, for example, FR-4 and Teflon for the microwave, high resistance silicon, quartz, and GaAs for the THz wave, and Ge for the infrared wave. In the first step, the material with high mobility for the cut-wire resonator was grown on the substrate. The material could be GaAs, InAs, Bi and graphene, and the method for the growing different material could include magnetron sputtering, molecule beam epitaxy (MBE), and chemical vapor deposition (CVD). Secondly, the cut-wire resonator would be patterned with lithography and dry etching. For the long wavelength metamaterial with large linewidth, such as microwave and THz waves, the standard ultraviolet lithography would satisfy the requirement. While for the short wavelength sample, such as infrared and visible light, the electron-beam lithography and focused ion beam (FIB) technology with nanoscale resolution might be involved. Thirdly, another lithography would be carried out to define the SRR, followed by the deposition of a highly conductive material with the techniques including sputtering and e-beam evaporation, and the material may include Au, Ag, Al and Cu. Finally, the lift-off process forms the SRR structure and completes the manufacturing.

FIGURE S9: **An exemplary fabrication process of the metamaterial. (a)** Growth of the high-mobility material for the cut-wire resonator. For different material, the method could be sputtering, MBE and CVD. **(b)** The cut-wire resonator was patterned by lithography and dry etching. For different wavelength, the lithography could be standard ultraviolet lithography, electron-beam lithography and FIB technology. **(c)** Second lithography and deposition of the highly-conductive material for the SRR. The deposition technique may be sputtering and e-beam evaporation. **(d)** A lift-off process to form the SRR structure.

[1] C. Lange *et al.*, "Extremely Nonperturbative Nonlinearities in GaAs Driven by Atomically Strong Terahertz Fields in Gold Metamaterials," *Physical Review Letters,* vol. 113, no. 22, p. 227401, Nov 28 2014.

[2] C. Vicario, M. Shalaby, and C. P. Hauri, "Subcycle Extreme Nonlinearities in GaP Induced by an Ultrastrong Terahertz Field," *Physical Review Letters,* vol. 118, no. 8, p. 083901, Feb 24 2017.

[3] M. van Exter and D. Grischkowsky, "Optical and electronic properties of doped silicon from 0.1 to 2 THz," *Applied Physics Letters,* vol. 56, no. 17, pp. 1694-1696, 1990.

[4] M. Ordal *et al.*, "Optical properties of the metals al, co, cu, au, fe, pb, ni, pd, pt, ag, ti, and w in the infrared and far infrared," *Applied Optics,* vol. 22, no. 7, pp. 1099-1119, 1983.

[5] S. Y. Tochitsky, R. Narang, C. Filip, C. Clayton, K. Marsh, and C. Joshi, "Generation of 160-ps terawatt-power CO 2 laser pulses," *Optics letters,* vol. 24, no. 23, pp. 1717-1719, 1999.
